# Supplementary material for: Pseudotyped virus infection of multiplexed ACE2 libraries reveals SARS-CoV-2 variant shifts in receptor usage
Source: PLoS Pathog. 2024 May 20;20(5):e1012044. doi: 10.1371/journal.ppat.1012044 (PMC11142672; doi:10.1371/journal.ppat.1012044)
Supplement: Attachment — Submitted filename: Response_to_reviewers.docx [file ppat.1012044.s013.docx]

**REVIEWER COMMENTS**

Reviewer 1, Major Issue #1: The expression level of each spike protein needs to be examined.

At the suggestion of the reviewer, we conducted an analysis of spike protein expression in both HEK293T producer cell lysates and cell supernatant obtained from these producer cells, following transfection with spike and lentivector reporter plasmids. To capture the pseudotyped virus particles, cell media was collected at 2, 3, and 4 days following transfection, and stored at 4°C until all samples were collected, pooled, and lysed. To capture the producer cell lysates, upon performing the last media collection, the remaining cells were collected and lysed. The lysates were separated via SDS-PAGE, and blotted with SARS-CoV-2 spike antibody, along with loading controls specific for cells and pseudotyped viral particles.

Using a SARS-CoV-2 spike S2 subunit -targeting antibody, we observed bands corresponding to full length and a cleaved S2 spike fragment for all samples, both in the cell lysate as well as in the produced particles. While there were slight differences in band intensities between samples, this appeared to be due to technical variability in transfection, as no variant spike was consistently higher or lower in intensity across the replicate experiments. The full-length Omicron BA1 spike, when present in the pelleted pseudotyped viral particles, consistently showed reduced migration through the polyacrylamide gel. As the Omicron BA1 spike encoded by our construct was the same polypeptide length or shorter than the other spike proteins we tested, this is likely due to Omicon-specific changes to spike protein molecular weight, likely from alterations in post-translational modification such as glycosylation.

Importantly, as the spike proteins were all shown to be expressed and incorporated into the pseudotyped viral particles, these Western Blotting experiments support the validity of the functional results yielded by our multiplex infection assay. We have included the Western Blotting images as a new Supplementary Fig S7. We have also included the experimental details in the materials and methods section of the manuscript, under the subheading “Immunoblotting of SARS-CoV-2 variant spike proteins on pseudotyped virus particles and cell lysates”.

Reviewer 1, Major Issue #2: Fig. 2A: the fold enrichment in the human ACE2 orthologs group is very small. How many independent experiments were performed? Were the differences statistically significant?

As noted in the third paragraph of the “Data analysis” subheading of the method section, “there were nine replicate infections and selections for D614G spike, five replicates for Alpha, two replicates for Beta, two replicates for Gamma, five replicates for Delta, and four replicates for Omicron BA1”. Since all samples were present in all replicates, this means that all ACE2 samples were replicated that many times for each infection condition. Since many of our analyses used the original, D614G spike as a reference, we oversampled those measurements to obtain the most accurate estimates of the true population mean as possible, for a total of nine. The high correlation we observed between the mean values from the two replicates of Beta with the mean values from the two replicates from Gamma suggested to us that further replicates were unlikely to be substantially more informative. For the others, an intermediate number of replication of four or five appeared sufficient for reasonable confidence in the ensuing results.

Regardless, the reviewer makes a great point that the human ACE2 mutants expressed at low abundance levels exhibit little magnitude difference between each other, and even small amounts of variability in the data could drastically affect the effect size and render the result statistically uninterpretable. We looked specifically at the statistical significance between the D614G and Omicron BA1 spike results, since the differential results between these spikes for the K31D, E35K, and D38H human ACE2 mutants are highlighted in the manuscript results section. We thus calculated the quotient when the enrichment scores for the human ACE2 variant in question was divided by the enrichment score for WT human ACE2, and plotted this for every replicate of D614G and Omicron BA1 spike pseudotype infection. For comparison, we also included E329K, which showed no effect on SARS-CoV-2 spike-mediated entry in a previous study, and also appeared to have no effect in this one.

The plot is now included as panel C of Supplementary Figure S1. The results show that, while the average values are indeed reflected by visually different populations of starting values, the differences are sufficiently small as compared to replicate variability that the differences would not reach statistical significance. While broader patterns involving multiple points will still be more statistically robust, this analysis highlights that individual data points with the human ACE2 mutants expressed at low levels should not be overinterpreted. We have included the following sentences in the manuscript to make this point:

“*Importantly, the fold enhancement of infection for low-abundance human ACE2 cells was quite low, and our current data was insufficiently powered to statistically analyze differences between the samples (Supplementary Fig S1C), which may indicate a limitation of using multiplex assays to study samples of relatively minor effect compared to other samples in the pool. Altogether, these results showed that our infection data can reveal interplays in molecular compatibilities that dictate ACE2 receptor usage by different SARS-CoV-2 variants, although broader patterns involving multiple points will still be more statistically robust than interpretations with specific data samples, particularly for those with low magnitudes of effect in the multiplex setting.*”

Reviewer 1, Major Issue #3: Fig. 5D: there seem to be big variations between the two repeats for human ACE2. Were the results for other ACE2 orthologs reproducible?

While there were some differences in SARS-CoV-2 spike variant infectivity between the two separate human ACE2-encoding samples, the differences were still minor when compared to the majority of the samples in the dataset, and their inclusion is helpful in denoting which other samples were largely indistinguishable for being human-like in our experiment.

Regardless, not including plots denoting the variability of the individual replicate measurements was an oversight in the original manuscript. While issues with data variability should be apparent with the averaged values used for the analyses and shown in the manuscript (eg. the high correlation between the results with the Beta and Gamma spikes would not be possible with problematically variable replicate data), visual representation of the non-averaged data is still an important part of understanding the interpretability of a given work. We have included a plot demonstrating the replicate score variability of D614G spike pseudotyped virus infection, represented by the enrichment score yielded by our DNA sequencing-based assay, as panel D in Supplementary Figure S1. We have also written the following sentence to the results section:

“*Importantly, the magnitude of infection enhancement observed between the samples within this dataset yielded effect sizes that clearly distinguished cells based on their infectability (Supplementary Fig S1D).*”

Reviewer 1, Minor Issue #1: Fig. 2A: please explain how the different level of ACE2 protein expression was achieved.

Thank you for making this request; our approach was described in detail within a previous publication (Shukla et al, PLoS Pathogens, 2021). Our intention was to avoid including a lengthy explanation in the results section of this manuscript, but we regret that a brief technical description was left out of the methods section, altogether. We have included a new paragraph within the “Plasmid construction, barcoding, and pooling” subsection of the materials and methods with the following text:

“*For the human ACE2 mutants expressed at low abundance levels, the “consensus” Kozak / translation initiation sequence of GCCACCATG preceding the protein coding sequence in the high abundance ACE2 ortholog constructs was replaced with the sequence “CATTGTATG”, which exhibits substantially reduced translation initiation, which we previously showed to cause a ~50-fold reduced steady-state abundance of the translated protein [3].”*

Reviewer 1, Minor Issue #2: Lines 390-392: these sentences are misleading. Natural infections occur with mixed populations of cells, but that doesn’t mean the receptors are different.

The point made by the reviewer is well taken; we have a separate study in progress where we study a mixed population of cells each expressing different levels of the same receptor, and this statement was meant for that study. We have removed this sentence from this manuscript. We did think there was still a related and factually correct statement that could be made, so we have replaced the original problematic sentence with the new one. The paragraph now reads:

“*The multiplexed target cell infection assay described in this work is a scalable cultured-cell model of viral entry that provides quantitative measurements of relative infectivities between samples. There are unique advantages this assay format possesses over traditional arrayed approaches. Having the full panel of receptors present within the same mixed pool of cells allows every sample to be exposed to the same inoculum of pseudovirus, minimizing inter-sample experimental variability. Highly infectable cells are unlikely to naturally exist as a monolayer, and their interspersal amongst poorly infectable cells within a multiplex assay approach may capture aspects of viral infection not normally encountered with assays with a clonal cell line. Our multiplexed assay format better recapitulates this situation, as arrayed approaches require different samples to be kept separate to maintain their identity.*”

Reviewer 1, Minor Issue #3: Fig. 10 and last paragraph in Discussion: the model that sustained transmission among humans leads to increased animal ACE2 compatibility seems to be highly speculative.

We agree that the model is speculative, but believe it is a worthwhile synthesis of the SARS-CoV-2 spike variant-specific ACE2 compatibility data generated in this work, with a vast literature of variant-specific antibody epitope disruption and evasion, already published by many other groups. Thus, to highlight how speculative the model is, we have explicitly written it into the title of Fig 10 and the last paragraph in the discussion.

Reviewer 2, Major Issue #1: The rationale for ortholog selection is unclear, however, and needs to be better defined. Of great interest would have been compatibility predictions for animal species in close contact with humans and species used frequently as animal models. If additional ACE2 orthologs were examined, inclusion of these data would substantially increase the impact of the manuscript.

The selection of orthologs and human mutants used in the work described in this manuscript was a completely practical one, as they were those that were already present in our lab from our previous work on SARS-CoV-2 entry, or those that were easily obtained through reagent sharing repositories such as Addgene. This is because the multiplex assay format described in this work was previously untested, and it is highly beneficial to use samples of known effect in other assay formats to benchmark a new one. Regardless, the benchmarking process itself yielded samples that could be easily studied in previously untested conditions; in this case, susceptibility to infection with the spike protein from the first series of SARS-CoV-2 spike variants of concern.

Importantly, there was a rationale for the study of the samples in the original study, and that relevance should, in many cases, transfer to this work. The human ACE2 mutants were those either shown or predicted to disrupt interaction with SARS-CoV or SARS-CoV-2 spike. The *Rhinolophus* horseshoe bat orthologs were previously created as part of a panel of diverse ACE2 sequences that represented potential hosts for distantly related sarbecoviruses; likewise, the *R. sinicus* alleles were chosen based on their high genetic distance from each other within the same species. The mouse, pig, and pangolin ACE2 sequences came from Professor Jeremy Luban’s lab via Addgene, and stemmed from their 2020 manuscript testing ACE2 with animal species which may have been associated with the SARS-CoV-2 pandemic. In aggregate, this diverse set of samples helped demonstrate the strengths and limitations of this assay format for studying virus entry.

We appreciate that such explanation is not made in the original version of the manuscript, and have included two additional sentences to the results section of the manuscript incorporating this. The whole paragraph now reads:

“*We appended unique barcode combinations to a set of 30 plasmids encoding two subsets of samples: ACE2 orthologs from a range of animals, including the reference sequence of human ACE2, and various mutants of human ACE2, with a subset known to affect SARS-CoV or SARS-CoV-2 entry [3] (Supplementary table 1). The ACE2 orthologs were largely from horseshoe bats of the genus Rhinolophus, originally chosen as a panel of diverse ACE2 sequences that represented potential hosts for distantly related sarbecoviruses. In aggregate, this diverse set of samples helped demonstrate the strengths and limitations of this assay format for studying virus entry. These ACE2 sequences were also previously studied with SARS-CoV-2 D614G spike using a traditional pseudotyped virus infection format, and were also easily obtained, making them convenient and effective benchmarks for the multiplex infection assay.*”

We completely agree with the reviewer that additional ACE2 orthologs, as well as compatibility predictions that could be made based on infection of a large set of ACE2 orthologs, would be extremely interesting. This is one of the current research directions in our research group, but we believe it would be thematically quite distinct from those posed by this current manuscript. Relatedly, we believe it would likely require an entirely new manuscript to perform the study in the most scientifically rigorous way.
